# Improvement of spinal non-viral *IL*-*10* gene delivery by D-mannose as a transgene adjuvant to control chronic neuropathic pain

**DOI:** 10.1186/1742-2094-11-92

**Published:** 2014-05-21

**Authors:** Ellen C Dengler, Lauren A Alberti, Brandi N Bowman, Audra A Kerwin, Jenny L Wilkerson, Daniel R Moezzi, Eugene Limanovich, James A Wallace, Erin D Milligan

**Affiliations:** 1 Department of Neurosciences, UNM School of Medicine, University of New Mexico Health Sciences Center, 1 University of New Mexico, Albuquerque, NM 87131-0001, USA; 2 Department of Anesthesiology and Critical Care Medicine, School of Medicine, University of New Mexico Health Sciences Center, 1 University of New Mexico, MSC10 6000, Albuquerque, NM 87106, USA; 3 Department of Neurosciences, Health Sciences Center, School of Medicine, University of New Mexico, Albuquerque, NM 87131-5223, USA

**Keywords:** M2 polarized, Cytokine, Interleukin-1β, Sciatic nerve, Immunofluorescence microscopy, Intrathecal injection, Dexamethasone, Allodynia, Rat

## Abstract

**Background:**

Peri-spinal subarachnoid (intrathecal; i.t.) injection of non-viral naked plasmid DNA encoding the anti-inflammatory cytokine, IL-10 (pDNA-IL-10) suppresses chronic neuropathic pain in animal models. However, two sequential i.t. pDNA injections are required within a discrete 5 to 72-hour period for prolonged efficacy. Previous reports identified phagocytic immune cells present in the peri-spinal milieu surrounding the i.t injection site that may play a role in transgene uptake resulting in subsequent IL-10 transgene expression.

**Methods:**

In the present study, we aimed to examine whether factors known to induce pro-phagocytic anti-inflammatory properties of immune cells improve i.t. IL-10 transgene uptake using reduced naked pDNA-IL-10 doses previously determined ineffective. Both the synthetic glucocorticoid, dexamethasone, and the hexose sugar, D-mannose, were factors examined that could optimize i.t. pDNA-IL-10 uptake leading to enduring suppression of neuropathic pain as assessed by light touch sensitivity of the rat hindpaw (allodynia).

**Results:**

Compared to dexamethasone, i.t. mannose pretreatment significantly and dose-dependently prolonged pDNA-IL-10 pain suppressive effects, reduced spinal IL-1β and enhanced spinal and dorsal root ganglia IL-10 immunoreactivity. Macrophages exposed to D-mannose revealed reduced proinflammatory TNF-α, IL-1β, and nitric oxide, and increased IL-10 protein release, while IL-4 revealed no improvement in transgene uptake. Separately, D-mannose dramatically increased pDNA-derived IL-10 protein release in culture supernatants. Lastly, a single i.t. co-injection of mannose with a 25-fold lower pDNA-IL-10 dose produced prolonged pain suppression in neuropathic rats.

**Conclusions:**

Peri-spinal treatment with D-mannose may optimize naked pDNA-IL-10 transgene uptake for suppression of allodynia, and is a novel approach to tune spinal immune cells toward pro-phagocytic phenotype for improved non-viral gene therapy.

## Background

Existing drugs, which primarily target neurons, partially reduce pain (by approximately 25 to 40%) in less than half of the 7 to 8% of patients suffering from chronic neuropathic pain in the US [1,2], which underscores the need to develop new therapeutic approaches to treat pathological pain. Modern views of pain processing are emerging which include critical roles of factors released from non-neuronal glial cells (microglia and astrocytes) in the central nervous system (CNS) [3-5], and satellite glia in the dorsal root ganglia (DRG) where sensory neurons are located [6,7]. Glial proinflammatory cytokines like IL-1β and TNF-α, and proinflammatory inducible factors such as calcium-independent nitric oxide (NO) are characterized to mediate the initiation and maintenance of experimental neuropathic pain [8]. Curiously, leukocytes (for example, macrophages, dendritic cells, T cells), responding to glial cytokines and NO, accumulate in DRG as well as in peri-spinal subarachnoid regions during neuropathies produced by remote, localized peripheral nerve lesions [9-13]. One possibility is that leukocyte-derived IL-1β, TNF-α, NO and other immune-related signaling factors can additionally contribute to feed-forward cytokine production and activity in the DRG and peri-spinal subarachnoid (intrathecal; i.t.) region. Notably, enrichment of leukocytes in these sites concomitant with neuropathic pain is generated in the absence of infection. Ultimately, the proinflammatory actions of spinal and DRG glia are capable of enhancing signals that mediate neuropathic pain in animal models.

The potent anti-inflammatory cytokine IL-10 can inhibit the actions of NO and a variety of cytokines including IL-1β and TNF-α by preventing intracellular kinase activation pathways, IL-1β and TNF-α protein production and release [14,15]. Additionally, IL-10 is a product of glia (astrocytes and microglia) [16] and leukocytes such as macrophages and dendritic cells, which express IL-10 receptors (see [17], for review). Importantly, adult spinal cord neurons do not naturally produce IL-10 and do not express IL-10 receptors, even under neuropathic conditions [18-20]. Prior reports demonstrate that IL-10 administration is an effective strategy to diminish pain-like behaviors in experimental animal models by blunting glial mediators of neuropathic pain signaling [20-25]. Thus, the application of IL-10 is a potential clinical therapeutic intervention of pathological cytokine and NO-mediated pain signaling that likely lacks direct actions on neurons.

Gene therapy has received some recognition as a treatment approach for pathological pain [26] with specific therapeutics targeting the actions of spinal glial cytokines [27]. While gene transfer using non-viral naked plasmid DNA (pDNA) is the least effective method to transform host cells with therapeutic genes of interest [28], prior work demonstrates that utilizing pDNA encoding the IL-10 transgene (pDNA-IL-10) when delivered via the i.t. route produces robust and sustained pain reversal in animal models [29,30]. Even though, a clearly defined mechanism by which spinal cord non-viral pDNA gains access to host cell machinery for transgene expression is poorly understood. However, one strong possibility is that gene transfection can result from non-specific phagocytosis by macrophages and dendritic cells [31] that have previously enriched the peri-spinal subarachnoid region. Although virtually every cell type is capable of phagocytosis, macrophages are highly specialized phagocytes that reside within healthy and intact peri-spinal meninges [32]. Additionally, microglia and astrocytes are ascribed as highly efficient phagocytic cells within the CNS [33,34]. Indeed, microglia are ‘the macrophages of the CNS’, and astrocytes maintain a healthy microenvironment by routinely clearing/digesting dying cells. Naked pDNA can stimulate macrophages, dendritic, and glial cells [35-38] to produce cytokines triggering leukocyte extravasation from circulation into peri-spinal meninges and the related i.t. space [30]. The meningeal phagocyte-enriched peri-spinal i.t. region may provide a key condition for augmented transgene uptake and expression. Different phenotypic activation profiles of immune and glial cells within the meninges may be particularly permissive for non-viral DNA-based gene transfer. Manipulating specific phenotypic activation profiles may be a unique approach to optimize transgene uptake resulting in long-duration therapeutic pain control. Specific activation of phagocytic leukocytes (particularly macrophages) leads to the production and release proinflammatory cytokines including IL-1β and TNF-α, characterized as generating an M1 polarized/classical activation phenotype (proinflammatory state) [39]. These same cell types have been identified as possessing a different activation profile, which is described as displaying pro-phagocytic properties with a distinct set of anti-inflammatory cytokines [40] like IL-4, IL-13 and IL-10 [41-44]. One hallmark of the anti-inflammatory activation profile is increased expression of the mannose receptor (MR [42] in the presence of increased IL-13, IL-4 and IL-10 protein levels with decreased IL-1β, TNF-α and NO production [39,41-44]. Moreover, the synthetic glucocorticoid, dexamethasone, is characterized to induce an M2 polarized/alternative activation phenotype with increased MR expression [42]. M2 polarized/alternatively activated leukocytes (for example, macrophages) and glial cells express low levels of proinflammatory IL-1β and TNF-α cytokines, and higher levels of IL-10 with enhanced IL-10-mediated phagocytic capacity [45]. Thus, spinal pDNA-IL-10 transgene uptake may be substantially improved by inducing an M2 polarized/alternatively activated phenotype in peri-spinal leukocytes.

**Scheme 1:**
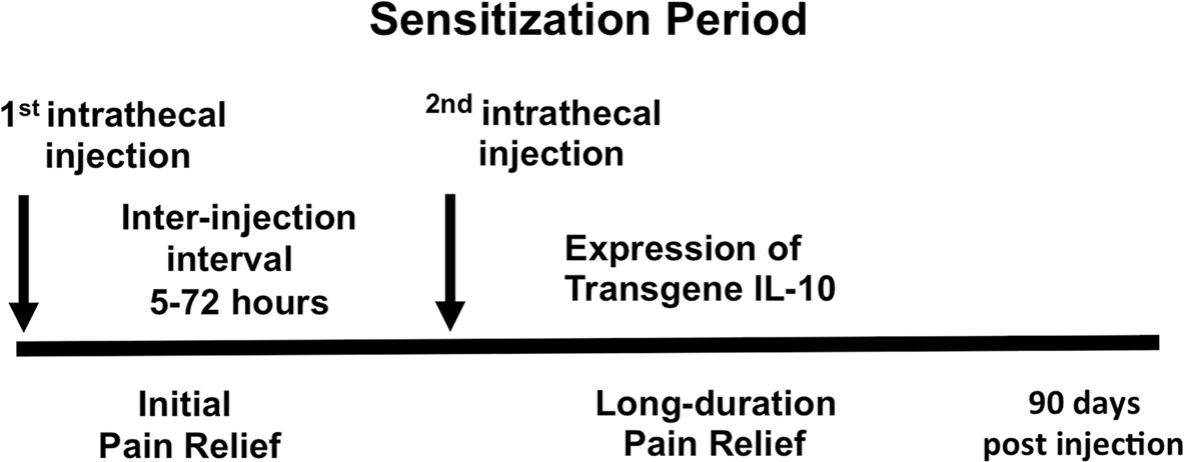
Depicting the ‘sensitization period’. **Depicting the ‘sensitization period’.** The scheme describes a discrete window of time characterized as a sensitization period that occurs from 5 to 72 hours following an i.t. pretreatment (also referred to as a first i.t. injection) of pDNA. During the sensitization period, an accumulation of leukocytes occurs surrounding the i.t. injection site. A possible mechanism underlying transgene therapeutic efficacy following this double-injection paradigm may be that accumulated peri-spinal leukocytes are induced to express an anti-inflammatory pro-phagocytic activation profile following the initial injection resulting in improved phagocytosis of subsequently delivered transgene.

We previously reported the discovery of a sensitization period between two necessary sequential peri-spinal i.t. injections of naked pDNA [29,30] producing long-duration pain reversal. While speculative, the sensitization period may involve the stimulation of peri-spinal phagocytic leukocytes (phagocytes) for improved pDNA-IL-10 transgene uptake (Scheme 1). In neuropathic rats, initial reversal from allodynia is observed following a first i.t. pDNA injection. Curiously, pDNA is not required to encode the *IL-10* gene used in the first injection. It is possible that the pDNA used for the first injection may act to stimulate surrounding phagocytes to (1) produce endogenous IL-10 protein and (2) maintain enhanced phagocytic efficiency prior to the second i.t. pDNA-IL-10 injection. The pDNA used for the second i.t. injection must encode the *IL-10* gene. Consequently, continual reversal of allodynia is observed for 90 days. Transgene-derived IL-10 mRNA expression is additionally observed early and long-after the second i.t. pDNA-IL-10 injection [46]. The sensitization period is discrete within the spinal i.t. microenvironment with a 5 to 72 hour inter-injection interval, as inter-injection intervals outside of this period fail to produce transgene-derived IL-10 mRNA expression, increased IL-10 protein production and long-duration pain relief [22,24,29,46]. Thus, peri-spinal phagocytes may participate in a local cellular response whereby enhanced uptake of pDNA-IL-10 [24] occurs within the sensitization period. The goal of the present studies is to determine whether inducing an M2 polarized/alternatively activated leukocyte/microglial profile improves pDNA-IL-10 uptake such that significantly lower transgene doses are efficacious for enduring pain relief. The critical time window to induce an M2 polarized/alternatively activated cellular profile is hypothesized to be within the sensitization interval. Dexamethasone and D-mannose, factors previously characterized to strongly induce anti-inflammatory M2-like alternative activation, are examined to generate improved pDNA-IL-10 transgene uptake, as assessed by the magnitude and duration of pain suppression and spinal IL-10 protein levels. Resultant behavioral profiles narrowed subsequent *in vitro* analysis of the optimal factor to be D-mannose. D-mannose pretreatment *in vitro* increases transgene-derived IL-10 protein release into cultured supernatants, with simultaneous decreases in IL-1β, TNF-α and NO. However, to provide parallel lines of evidence for targeting an M2-like phenotype, examination with IL-4 pretreatment was also studied as a gene therapy adjuvant. IL-4 has been shown to reduce spinal neuroinflammation and suppress cancer pain [47]. Yet, *in vitro* IL-4 pretreatment did not result in enhanced transgene uptake. Lastly, *in vivo*, D-mannose facilitates therapeutic efficacy of spinal pDNA-IL-10 with simultaneous increases in IL-10 protein expression in discrete spinal and DRG regions critical for sensory processing. Together, these data suggest that D-mannose is capable of optimizing transgene uptake engaging cellular mechanisms that are potentially M2-independent.

## Materials and methods

### Animals

A total of 112, adult, male Sprague-Dawley rats (Harlan Labs, Houston, TX, USA), 300 ± 5 g, were used in these experiments and were pair-housed housed at 21 ± 2°C in light controlled rooms (12:12 light:dark; lights on at 6:00 am) fed standard rodent chow and water available *ad libitum*. Behavioral testing was performed during the first three hours of the light cycle. All procedures were approved by the Institutional Care and Use Committee (IACUC) of the University of New Mexico following NIH Guidelines for the Care and Use of Laboratory Animals, and closely adhered to guidelines from the International Association for the Study of Pain for the use of animals in research.

### von Frey test for mechanical allodynia

The von Frey test was used to quantify rat hindpaw responses to tactile stimulation and conducted identically as previously described [48,49]. Briefly, rats were habituated to the testing environment consisting of a quiet and dimly lit room using red lighting where rats were placed on an overhead shelf composed of open powder-coated wire grid (inter-bar space; 8 mm) allowing full access to the entire plantar surface of the hindpaw for tactile stimulation using a logarithmic series of calibrated Semmes-Weinstein monofilaments (North Coast Medical, Inc. Gilroy, CA, USA). Rats were habituated for 4 consecutive days, 20 to 30 minutes/day in the testing environment at 26 to 27°C. Following habituation, baseline (BL) hindpaw responses were assessed using the monofilaments. Ten monofilaments were used, each with a log-stiffness value, defined as log_10_ (mg × 10); values in grams follow in parentheses: 3.61 (.407), 3.84 (.692), 4.08 (1.202), 4.17 (1.479), 4.31 (2.041), 4.56 (3.630), 4.74 (5.495), 4.93 (8.511), 5.07 (11.749) and 5.18 (15.136). At each trial, the following protocol was used. Beginning with the 4.31-labeled monofilament, rat hindpaws were stimulated three times. If hindpaw responses occurred ≥ 33% of the stimulus presentations with the 4.31 monofilament, then the lightest monofilament (3.61) was used for the next stimulus. Stimulus presentations continued using increasingly heavier monofilaments (for example, 3.84, 4.08, and so on) and were terminated upon a 100% (3/3 trails) hindpaw response rate. However, if hindpaw responses occurred ≤ 33% of the stimulus presentations with the 4.31 monofilament, then the next heavier monofilament (4.56) was used. Stimulus presentations continued with increasingly heavier monofilaments (for example, 4.74, 4.93, and so on), and presentations were terminated upon a 100% (3/3 trails) hindpaw response rate. This approach streamlines the total time for threshold assessment and avoids overstimulation of the hindpaws at supra-threshold levels. The monofilaments were applied perpendicular to the hindpaws for eight seconds to determine the threshold stiffness that would elicit paw withdrawal. Both ipsilateral and contralateral withdrawal responses were assessed and testing was conducted prior to BL and three and ten days after surgery, and again following i.t. injections at designated time points as indicated in the representative figures. Three BL measures were averaged for the right and left paws separately. The log stiffness that resulted in the 50% response threshold values (absolute threshold) was computed by fitting to a Gaussian integral psychometric function, PsychoFit, allowing parametric statistical analysis [49-52]. The data are presented graphically as ‘Absolute Log Stimulus Intensity (mg × 10)’ with a corresponding linear scale from 0.32 to 15 g. The software for PsychoFit may be downloaded from LO Harvey’s website (http://psych.colorado.edu/~lharvey).

### Chronic constriction injury (CCI)

The surgical procedure for chronic constriction injury (CCI) was performed similarly as previously described [53,54]. Briefly, under isofluorane anesthesia (1.5 to 2.0% volume in oxygen), the mid to lower back and dorsal thigh were shaved and cleaned with diluted Bactri-Stat AE (EcoLab Health Care Division, Mississauga, Ontario, Canada). Using aseptic procedures, one sciatic nerve was carefully isolated using sterile glass curved probes, followed by snugly tying four 4-0 chromic gut sutures (Ethicon, Somerville, NJ, USA) around one sciatic nerve while ensuring the suture material did not pinch into the underlying nerve. The overlying muscle was sutured closed with two sterile silk sutures (Ethicon, Somerville, NJ, USA), and the overlying skin was closed with three or four wound clips. The sciatic nerves of sham-operated rats were identically exposed but not ligated. Following surgical procedures, rats were placed in a heating chamber, with temperature maintained at 27°C, and fitted with a clear plastic perforated lid to observe rats upon recovery from anesthesia. Full recovery from anesthesia was observed within ten minutes, and rats that had undergone CCI showed minor ventroflexion of the ipsilateral hindpaw, while rats with sham surgery revealed no change from pre-surgical manipulation in hindpaw posture. Animal body weight was recorded following surgery. All rats were additionally monitored for five minutes one day following surgical manipulation. During inspection, the rat’s gait, the affected hindpaw’s posture, the condition of the hindpaw skin, and activity level was noted. In these studies, < 1% of rats with CCI revealed unusual hindpaw ventroflexion with the presence of autotomy, and were immediately euthanized.

### Drugs

Commercially available water soluble dexamethasone (DEX; catalog # D2915) and D-mannose (catalog # M6020) were purchased from Sigma-Aldrich (St. Louis, MO, USA). IL-4 was purchased carrier free with 0.1% BSA added upon reconstitution in sterile saline (R&D Systems Inc., Minneapolis, MN, USA). The vehicle for both drugs was isotonic sterile saline (catalog # 7199, Physician Sales & Services, Grand Prairie, TX, USA).

### Preparation of plasmid DNA

The plasmid vector used in these studies is fully described previously [29]. It consists of a 5.9 Kilobase (Kb) circular plasmid DNA containing a transcriptional cassette consisting of a cytomegalovirus enhancer/chicken beta-actin promoter (CMV enh/CB pro) driving expression of the rat *IL-10* gene containing a point mutation (F129S) and a viral SV40 polyadenylation signal. The transcription cassette is flanked by a 149 bp inverted terminal repeat sequence. An identical plasmid lacking the *IL-10* gene was used as a pDNA control. Both plasmids were amplified in SURE 2 Supercompetent *E. coli* cells (Agilent Technologies, http://www.genomics.agilent.com., USA) and isolated using an endotoxin free Giga plasmid purification kit (Qiagen, Valencia, CA, USA) according to the manufacturer’s instructions. Purified, endotoxin-free plasmids were resuspended in sterile Dulbecco’s PBS (DPBS, 1, 0.1 micron pore-filtered, pH 7.2, catalog # 14190-144; Gibco, Invitrogen Corp, Grand Island, NY, USA) with 3% sucrose (DPBS-3%). The DPBS-3% vehicle was prepared using molecular biology grade D (+)-sucrose (b-D-fructofuranosyl-a-D-glucopyranoside; Sigma-Aldrich, St. Louis, MO, USA) in DPBS, 0.2 um sterile filtered (pyrogen-free syringe filter unit, catalog # 25AS020AS, Life Science Products, Inc., CO, USA) and stored in sterile, 15 ml conical tubes at 4°C until the time of use.

### Intrathecal (i.t.) injections

Injections were acutely administered and conducted as described previously [29]. Briefly, rats were anesthetized with isofluorane (5% volume in oxygen) and an 18-gauge guide cannula constructed from an 18-gauge, 4.5 cm length sterile hypodermic needle (Beckton Dickinson & Co., Franklin Lakes, NJ, USA) with the plastic hub removed, was inserted percutaneously between lumbar vertebrae 5 and 6 (L5-6). During this time, a slight tail flick followed by a small amount of cerebrospinal fluid (CSF) efflux from the 18-gauge cannula was observed, indicating subarachnoid catheter placement.

Injectors used for i.t. delivery were constructed as follows. A 50 μl sterilized Hamilton syringe connected to a 45 cm-length polyethylene tubing (PE-10; catalog # 427401; Becton Dickinson, Sparks, MD, USA) via a 30-gauge, 0.5-inch needle inserted into one end of the PE-10 tubing with the hub connected to the Hamilton syringe. The open end of the PE-10 tubing (catheter) was then marked at 7.7 cm using a black permanent marker, and the open end was used to draw 1 μl air followed by 20 μl of drug. The drug-filled PE-10 catheter was then inserted into the open end of the L5-L6-placed 18-gauge guide cannula and advanced 7.7 cm rostrally, placing the internal portion of the PE-10 catheter over the L4-L6 lumbosacral spinal cord enlargement where axon terminals of sciatic afferent nerve fibers synapse onto spinal cord dorsal horn neurons. Injections were given over a 0.5 to 1 minute interval. Following drug injection, the PE-10 catheter and the 18-gauge cannula were immediately removed and discarded. The total time required for these injections was two to three minutes. Following recovery from anesthesia, 100% of the animals resumed motor activity consistent with that observed prior to the i.t. injection. To evaluate this, rats were placed on a table top and observed for two minutes and gait, ipsilateral and contralateral hindpaw posture and tail movement were observed.

### Immunohistochemical tissue sample preparation

Procedures described for tissue processing closely followed those previously described [55,56]. Briefly, following behavioral assessment at day 29 after i.t. injection, animals were overdosed with sodium phenobarbital (Sleepaway, Fort Dodge Animal Health, Fort Dodge, IA, USA) and perfused transcardially with 0.1 M PBS (pH 7.4) followed by 4% paraformaldehyde (pH 7.4). Whole vertebral columns with intact spinal cords (cervical 2 through sacral 1 spinal column segments) were removed, and underwent overnight fixation in 4% paraformaldehyde at 4°C. This procedure ensured that spinal cord, DRG, and related meninges, were anatomically intact with respect to each other. All specimens underwent ethylenediaminetetraacidic acid (EDTA; catalog # EDS; Sigma Aldrich, St. Louis, MO, USA) decalcification for 30 days, and spinal cord sections were subsequently paraffin processed and embedded in Paraplast Plus Embedding Media (McCormick Scientific, St. Louis, MO, USA), as previously described [57]. Adjacent tissue sections (7 μm) were mounted on vectabond-treated slides (Vector Labs, Burlingame, CA, USA), and allowed to adhere to slides overnight at 40°C, followed by deparaffinization, and rehydration via descending alcohols to PBS (1×, pH 7.4). Sections were then processed with microwave antigen retrieval procedures (citrate buffer pH 6.0, or tris-based buffer, pH 9.0; BioCare Medical, Concord, CA, USA).

### Antibody staining

Procedures conducted for antibody staining were similar to that previously described [55,56]. Briefly, slides were incubated with 5% normal donkey serum (NDS), in PBS (1×, pH 7.4) for two hours, followed by overnight primary antibody incubation in a humidity chamber at 4°C. Slides underwent secondary antibody incubation for two hours in a humidity chamber at room temperature, rinsed in PBS, and then coverslipped with Vectashield containing the nuclear stain 4′,6-diamidino-2-phenylindole (DAPI) (Vector Labs, Burlingame, CA, USA). Primary antibodies to detect IL-10 (R&D Systems Inc,, Minneapolis, MN, USA) and IL1-β (Santa Cruz Biotechnology, Santa Cruz, CA, USA), expression were used on tissue sections that were incubated overnight followed by incubation with biotinylated secondary antibody for one hour, and then treated with Vectastain ABC Elite kit (Vector Labs, Burlingame, CA, USA) and subsequently stained using TSA Plus Fluorescein System (PerkinElmer Life Sciences, Waltham, MA, USA), and finally cover slipped with Vectashield containing DAPI. For lumbar spinal cord, sections ipsilateral to sciatic nerve CCI were taken from L4-L6, and corresponding L5 DRG sections were examined with the most proximal portion of the DRG analyzed.

### Spectral imaging for immunofluorescent quantification

Procedures conducted for immunofluorescent quantification of stained sections were performed similarly to that previously described [55,56], with the following conceptual clarification. Briefly, spectral image acquisition (420 to 720 nm) of tissue was obtained using an Axioscope microscope connected to a Nuance Camera 2.8 (FX) Multispectral Imaging System, (PerkinElmer, Waltham, MA, USA). This camera contains a liquid crystal tunable filter (LCTF) able to filter light with a range of 420 to 720 nm at 10 nm increments generating a series (30) of fluorescent intensity profiles (420 to 720 nm) within a discrete area (1 pixel). There are 512 × 512 pixels that create the entire area of tissue being analyzed. Thus, the entire spectral information (30 profiles that represent 420 to 720 nm emission) are contained within each pixel, and a total of 262,144 pixels are analyzed by the by CRI software (Cambridge Research and Instrumentation Inc., (CRI) Wolburn, MA, USA) to determine the pattern of spectral intensities from 420 to 720 nm (peak and low level intensities) for a particular tissue specimen. These data represent the unique pattern that a tissue will display when stained, for example, with a Rhodamine Red or FITC-green secondary antibody, thus allowing the user to distinguish between tissue autofluorescence and true secondary antibody staining. A major aspect of the software allows the user to subtract background fluorescence from the image being analyzed. Background is defined as any spectral emission pattern that is different from the unique pattern generated by secondary antibody. Images were de-convoluted into images containing: 1) the spectra of a true secondary antibody fluorescence (for example, Rhodamine Red emission), as determined from a control cover-slipped slide on which a small drop of 100x diluted fluorophore-conjugated secondary antibody was placed, and 2) the pattern of autofluorescence wavelength emission-spectra determined for spinal cord and DRG tissue not exposed to primary or secondary antibody. Two sets of control slides with tissue sections were acquired; one with PBS without primary but containing secondary antibody treatment, and the other with primary but without secondary antibody treatment. These controls slides were then used to objectively eliminate autofluorescence and artifactual very low-intensity fluorescence. Using these control slides, the Nuance software allows the user to set an acceptable threshold of very low-level emission fluorescent intensity (as opposed to the software’s ‘autothreshold’ option). The experimenter determines artifactual low-level emission intensity by closely replicating the composite computer image with that observed through the microscope eyepiece. The resultant image contains Rhodamine Red without very low-level emission intensity and without autofluorescence while retaining all of the cellular and anatomical features of the actual tissue specimen.

The magnitude of fluorescent wavelength signal was calculated by the computer software for each area of contiguous pixels defined as a region of interest (ROI). The Rhodamine Red fluorescent signal intensity for each ROI was quantified and given a numerical value. These signal ‘counts’ were then averaged and divided by the exposure time for each image collected per ROI. An image was captured for each of 4 slices (n = 4 slices per rat) with 3 rats per group (n =12 total slices analyzed per experimental group) for the spinal cord, and separately for the DRG (ipsilateral). The positive Rhodamine Red data from all 12 slices per experimental group were then averaged to represent a group average and reported as, ‘signal counts/sec/mm^2^’. Averages are reported with the group variance (standard error). More detailed information regarding the Nuance spectral system can be found at URL: (http://www.cri-inc.com/products/nuancew.asp).

### Cell culture

Mouse macrophage (RAW 264.7) cells were obtained from American Type Culture Collection (catalog # TIB-71; ATCC Manassas, VA, USA) and cultured as adherent cells in DMEM (catalog # D6429; Sigma-Aldrich, St Louis, MO, USA) supplemented with 10% heat-inactivated FBS (catalog # 10082-147; Gibco-Life Technologies, Grand Island, NY, USA) and 100 U/ml penicillin with 100 μg/ml streptomycin (catalog # 15140122; Gibco-Life Technologies, Grand Island, NY, USA). Cells were maintained at 37°C under humidified 5% CO_2_ atmosphere. Cells were grown to 85% confluency, collected by scraping, and sub-cultured for three passages. Dead cells were identified by using trypan blue exclusion.

### Nitric oxide assay

RAW 264.7 cells were seeded at a density of 3.0 × 10^5^ cells/ml in 24-well plates 24 hours prior to experimentation and maintained at 37°C under a humidified 5% CO_2_ atmosphere. At 85% confluence, the supernatant (1 ml/well) was removed and replaced with DMEM containing 10 mM mannose or fresh DMEM that was allowed to incubate for one hour. After one hour incubation with media alone or D-mannose (10 mM), media from the wells was removed and replaced with fresh DMEM containing either, 10 ng/ml lipopolysaccharide (LPS) from *E. coli* (LPS; catalog # L6529; Sigma-Aldrich, St Louis, MO, USA), LPS (10 ng/ml) plus D-mannose (10 mM), or DMEM alone. The different treatments were allowed to incubate with the cells for 10 minutes, followed by removal of supernatant and a 2× wash with 1× PBS (pH 7.4, catalog # 10010; Gibco-Life Technologies, Grand Island, NY, USA). Supernatant was removed from each well and NO production was measured using commercially available Griess Reagent System following the manufacturer’s instructions (catalog # G2930; Promega Corp., Madison, WI, USA). Briefly, 50 μl of supernatant from each well was removed and mixed with 50 μl of sulfanilamide solution and 50 μl of N-1-napthylethylenediamine dihydrochloride. The reaction was allowed to incubate protected from light and the absorbance was measured at 550 nm using a Tecan Infinite® plate reader (Tecan Systems, Inc., San Jose, CA, USA). Limit of detection for the NO assay = 1.56 μM. All experiments were run with triplicate biological samples.

### Quantification of IL-1β, IL-10, TNF-α protein levels

RAW 264.7 cells were treated exactly as outlined above with the exception that 100 mM D-mannose was used in the appropriately designated treatment group. D-mannose was incubated for two hours with LPS. At 24 hours, supernatant was collected and species-specific mouse IL-10, IL-1β, TNF-α, IL-4, and rat IL-10 were assayed via ELISA according to manufacturer’s instructions (IL-1β, catalog # SML800C; IL-4, catalog # M4000B; mIL-10, catalog # M1000; rIL-10, catalog # R1000; TNF-α, catalog # MTA00B, R&D Systems Inc., Minneapolis, MN, USA). Limit of detection for the IL-10 protein assays = 15.6 pg/ml; positive IL-10 kit control value = 117.68 pg/ml; limit of detection for the IL-1β assay = 12.5 pg/ml; positive IL1-β kit control value = 553.75 pg/ml; limit of detection for the TNF-α = 10.9 pg/ml; positive kit control value = 110.17 pg/ml.

In a separate experiment, cells were pretreated with either IL-4 (100 ng/ml) with 0.1% BSA for 24 hours, as established previously [58,59], or 500 mM D-mannose for five hours or media alone. The media was then removed and replaced with fresh DMEM containing either pDNA encoding rat IL-10 (0.5 or 5.0 μg/μl) or media alone. All experiments were run according to manufacturer’s instructions and with triplicate biological samples.

### Data analysis

Behavioral analysis for hindpaw threshold responses was performed as previously described [49] to compute the log stiffness that would have resulted in the 50% paw withdrawal rate. Briefly, thresholds were estimated by fitting a Gaussian integral psychometric function to the observed withdrawal rates for each of the tested von Frey hairs, using a maximum-likelihood fitting method [50,52]. Estimated thresholds derived from a Gaussian integral function yield a mathematical continuum and thus, are appropriate for parametric statistical analyses [49,50,52]. The computer program PsychoFit may be downloaded from LO Harvey’s website (http://psych.colorado.edu/~lharvey). At baseline and prior to surgical manipulations, one-way analysis of variance (ANOVA) procedures were applied to identify potential group differences of hindpaw threshold values. Repeated measures two-way ANOVA procedures were applied to determine statistical significance between experimental treatment groups, with significance determined at *P* < 0.05. All other data analyses were performed using one-way ANOVA. All statistical analysis was performed on the computer program GraphPad Prism version 4.03 (GraphPad Software Inc., San Diego, CA, USA). All data are expressed as mean ± SEM. For *post hoc* analysis, the Bonferroni’s test was performed. Given that unusually high variance was observed in immunofluorescent-quantified tissue only from the saline-pDNA-IL-10 group, Grubbs’ *Z*-test to identify outliers [60,61] was applied.

## Results

### Dexamethasone pretreatment followed by i.t. pDNA-IL-10 produces transient and minimal behavioral reversal of allodynia

**Figure 1:**
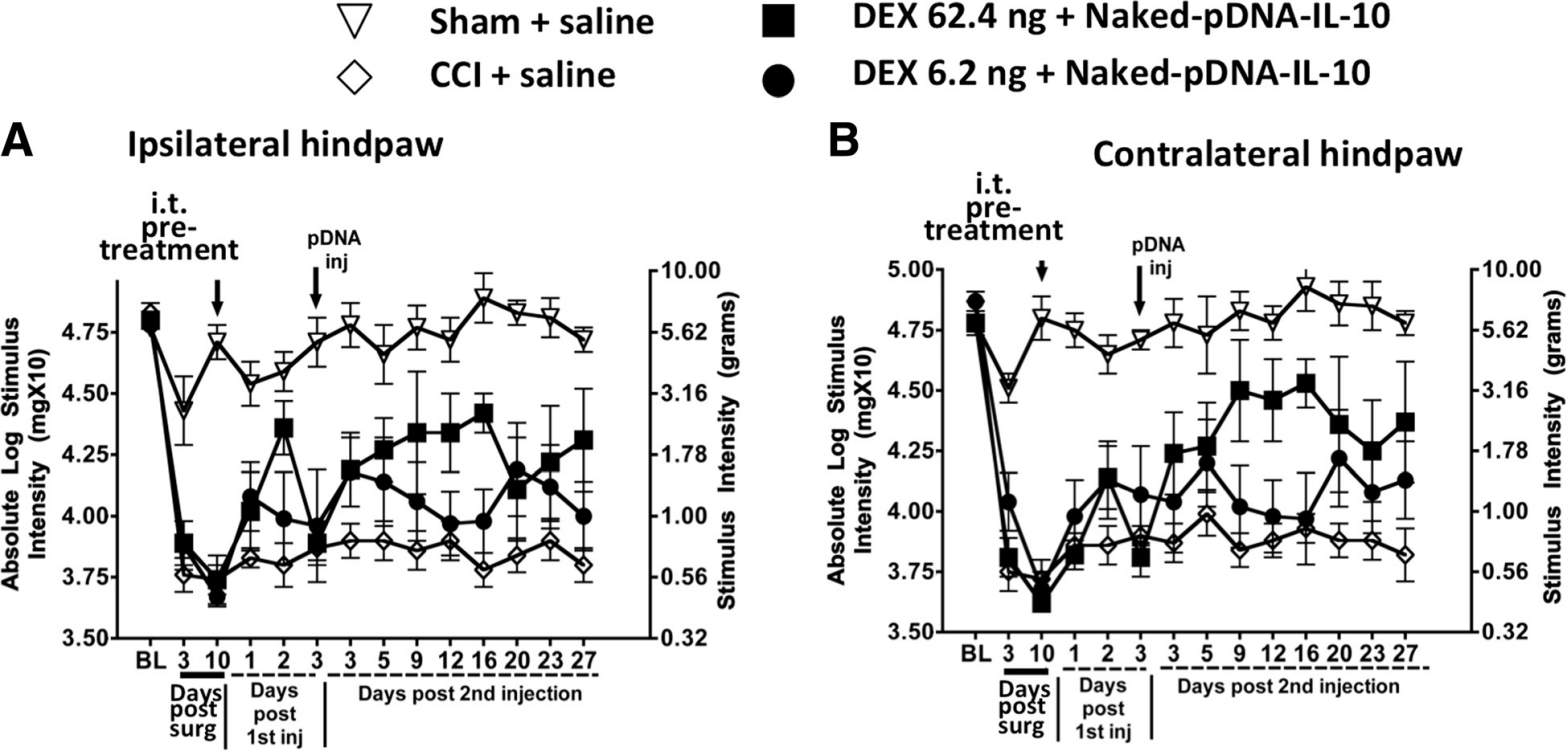
**Dexamethasone for improved pDNA-IL-10 uptake does not create robust pain reversal. (A and B)** animals developed stable allodynia from day 3 to day 10 after chronic constriction injury (CCI), compared to sham-operated animals (ipsilateral, F_(1, 20)_ = 53.54; *P* < 0.0001; contralateral, F_(1, 20)_ = 71.70; *P* < 0.0001). On day 10 following CCI surgery, animals received an i.t. injection of dexamethasone (DEX) (62.4 ng, n = 6 or 6.2 ng, n = 6), or equivolume i.t. saline (n = 7) and sham-operated animals received i.t. equivolume saline (n = 6). Three days later, an i.t. injection of pDNA-IL-10 (25 μg) or equivolume saline was given. I.t. pDNA-IL-10 following a pre-injection injection of DEX (62.4 ng) revealed a delayed and partial bilateral pain reversal (ipsilateral, F_3,140_ = 33.83; *P* < 0.0001; contralateral, F_3,140_ = 19.7; *P* < 0.0001). Black arrows indicate i.t. injections.

The synthetic glucocorticoid, dexamethasone (DEX), given as an i.t. pretreatment followed by i.t. pDNA-IL-10 (25 μg) results in a transient reversal of allodynia produced by chronic constriction injury (CCI) of one sciatic nerve. CCI is induced by chromic gut suture loose-ligation of the intact sciatic nerve leading to local inflammation plus slight constriction. This procedure results in clinically relevant neuropathic behavioral changes such as allodynia, as assessed by sensitivity to light touch using the von Frey test. Baseline hindpaw sensory threshold responses to light mechanical touch measured by the von Frey test with calibrated monofilaments revealed similar levels between all groups, as no significant differences were observed (ipsilateral, F_(3,24)_ = 0.2154; *P* > 0.8; contralateral, F_(3,24)_ = 0.6930; *P* > 0.5). Following either CCI or sham surgery, behavioral testing continued at the time points indicated (Figure 1). Compared to sham-operated controls that reveal stable threshold responses near BL values, clear development of allodynia on the side ipsilateral and contralateral to sciatic nerve manipulation is observed in CCI-operated rats three and ten days after CCI, with responses now occurring at < 1.0 g of touch stimuli (Figure 1). Beginning on day 10 and throughout the 27-day timecourse, sham- or CCI-treated rats given an i.t. pretreatment of saline followed three days later with i.t. saline reveal stable hindpaw responses similar to BL values, or < 1.0 g, in sham or CCI animals, respectively. Paw withdrawal thresholds in those CCI animals given i.t. pretreatment of 62.4 ng DEX followed three days later by i.t. pDNA-IL-10 (25 μg) revealed a delayed partial and short-term reversal of allodynia, lasting only two weeks (Figure 1). I.t. pDNA-IL-10 following pretreatment with DEX (62.4 ng) revealed a delayed and partial bilateral pain reversal (ipsilateral, F_3,140_ = 33.83; *P* < 0.0001; contralateral, F_3,140_ = 19.7; *P* < 0.0001). No significant threshold changes from allodynia were observed throughout the 27-day timecourse in rats given an i.t. pretreatment of 6.2 ng DEX followed three days later with i.t. naked pDNA-IL-10.

### D-mannose pretreatment improves low-dose pDNA-IL-10 efficacy for long-term reversal from allodynia

**Figure 2:**
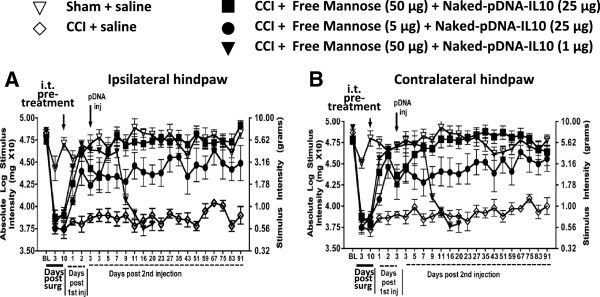
**D-mannose improves pDNA-IL-10 anti-allodynia efficacy for 90 days. (A and B)** sham operated animals (open triangles; n = 6) versus chronic constriction injury (CCI) animals (open diamonds; n = 7) whereby allodynia persisted to day 10 (ipsilateral, F_(4,28)_ = 32.87; *P* < 0.0001; contralateral, F_(4,28)_ = 37.01; *P* < 0.0001). On day 10, animals received an i.t. pretreatment of either D-mannose (50 μg; 5 μg), or equivolume saline followed three days later by pDNA-IL-10 (25 μg or 1 μg) or equivolume saline. Following pretreatment with D-mannose (50 μg; closed squares; n = 8), reversal of hindpaw thresholds was observed compared to CCI-saline, or CCI D-mannose (5 μg) injection (ipsilateral, F_(4, 70)_ = 15.87; *P* < 0.0005; contralateral, F_(4, 70)_ = 20.40; *P* < 0.001). Full reversal continued for a three-month period beyond the second injection of pDNA IL-10 (25 μg) in those animals given D-mannose (ipsilateral, F_(3, 384)_ = 57.46; *P* < 0.0001; contralateral, F_(3, 384)_ = 59.20; *P* < 0.0001). Animals pretreated with the lower dose of D-mannose (5 μg) followed by a second injection of pDNA-IL-10 (25 μg) (closed circles, n = 7) showed partial bilateral reversal. A second injection of a lower dose of pDNA-IL-10 (1 μg: closed triangles; n = 5) produced a transient 11-day reversal compared to other CCI + D-mannose treated rats (ipsilateral, F_(3.17)_ = 12.71; *P* < 0.03; contralateral, F_(3, 17)_ = 14.64; *P* < 0.005).

An i.t. pretreatment of D-mannose dose-dependently and dramatically increased the efficacy of pDNA-IL-10 to reduce allodynia (Figure 2). At BL, all groups revealed similar hindpaw threshold responses as determined by the von Frey test (ipsilateral, F_(4,28)_ = 1.009; *P* > 0.4; contralateral, F_(4,28)_ = 1.147; *P* > 0.3). Non-neuropathic sham-operated i.t. saline injected rats revealed stable basal responses while CCI-treated i.t. saline injection revealed chronic bilateral allodynia throughout a 91-day timecourse. Strikingly, pretreatment with i.t. D-mannose (50 μg) followed three days later by i.t. pDNA-IL-10 (25 μg) produced a complete three-month reversal of allodynia with hindpaw responses occurring at levels similar to BL thresholds. Curiously, even a ten-fold lower i.t. dose of of D-mannose (5 μg) followed by i.t. pDNA-IL-10 (25 μg) produced a partial and enduring relief from bilateral allodynia. Furthermore, i.t. pretreatment with D-mannose (50 μg) followed three days later by a 25-fold lower dose of i.t. pDNA-IL-10 (1 μg) resulted in a complete, albeit brief, nine-day reversal from allodynia (Figure 2).

### D-mannose generates short-term allodynia reversal and anti-inflammation

**Figure 3:**
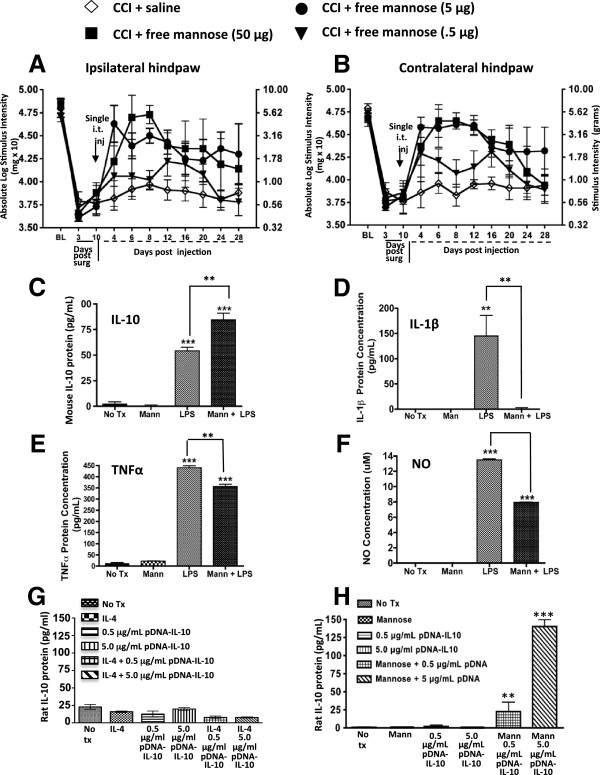
**D-mannose generates short-term reversal of allodynia, an M2 polarized phenotype and improved pDNA-IL-10 transgene uptake in macrophage cell cultures. (A and B)** after baseline (BL) assessment, all animals underwent chronic constriction injury (CCI) surgery resulting in allodynia by day 10. On day 10, animals were given a single i.t. injection of D-mannose (50, 5, or 0.5 μg; closed squares, closed circles, or closed diamonds respectively) or equivolume saline (n = 3 to 4 per group). Saline-treated animals (open diamonds) remained bilaterally allodynic. Treatment with D-mannose (50 μg, n = 3) resulted in bilateral partial reversal from allodynia that returned by day 20 following i.t. injection (ipsilateral, F_(3,70)_ = 15.87; *P* < 0.0005; contralateral F_(3,70)_ = 20.40; *P* < 0.0001). Black arrows indicate i.t. injection. **(C-F)** cultured Raw 264.7 mouse macrophage cells were pretreated with D-mannose (100 mM) followed by a two-hour incubation with a combination of D-mannose (100 mM) and lipopolysaccharide (LPS) (10 ng). **(C)** compared to control treatment (No Tx = no treatment; Mann = D-mannose), LPS-stimulated cells given D-mannose treatment resulted in significantly increased mouse IL-10 protein levels, **(D)** almost complete ablation of IL-1β levels, **(E)** significantly reduced TNF-α protein levels, and **(F)** reduced NO production. **(G)** cultured Raw 264.7 mouse macrophage cells treated with IL-4 did not improve pDNA-IL-10 transgene expression, however, **(H)** D-mannose generated a robust dose-dependent increase in pDNA-IL-10 transgene expression. *P < 0.05; **P < 0.01; ***P < 0.0001.

The above-described data show the effects of D-mannose pretreatment on the pain therapeutic efficacy of reduced pDNA-IL-10 doses. Notably, D-mannose pretreatment facilitates low-dose (1 μg) pDNA-IL-10 effects on pain reversal, albeit transient. However, D-mannose may exert enduring pain-suppressive effects in the absence of the IL-10 transgene. To examine this question, we investigated whether a single i.t. injection of D-mannose could dose-dependently sustain prolonged pain relief. BL response thresholds were similar between groups prior to CCI (ipsilateral, F_(3,13)_ = 0.4499; *P* > 0.5; contralateral, F_(3,13)_ = 0.1761; *P* > 0.90). However, threshold responses revealed clear bilateral allodynia three and ten days later (Figure 3A and B), with no significant differences between groups (F_(3,13)_ = 0.1897; *P* > 0.90; contralateral, F_(3,13)_ = 0.2234; *P* > 0.87). On day 10, a single i.t. injection of 50, 5, or 0.5 μg D-mannose or equivolume saline was delivered. Behavioral thresholds revealed a robust transient bilateral reversal from allodynia following 50 or 5 μg D-mannose compared to either 0.5 μg D-mannose or saline (Figure 3A and B).

One potential mechanism by which D-mannose leads to the observed transient reversal from allodynia is via generating an anti-inflammatory response. To discretely examine this possibility, mouse macrophage RAW 264.7 cell cultures were used because macrophages are present in the peri-spinal subarachnoid region [32], and these immune cells are professional phagocytes. A classic proinflammatory response was initiated by lipopolysaccharide (LPS), which consists of cell-wall particles from gram-negative bacteria. Induction of the proinflammatory environment mimics the local peri-spinal signaling milieu present during chronic peripheral neuropathic conditions. Such signaling molecules present during proinflammatory conditions include increased TNF-α, IL-1β and NO production.These data reveal that cells treated with LPS induced classic macrophage responses as measured by increased IL-10, IL-1β and TNF-α protein levels with concurrent increases in NO production (Figure 3C-F). However, pretreatment with D-mannose generated additional IL-10 protein increases (Figure 3C) with simultaneous ablation of IL-1β protein (Figure 3D), and a robust reduction of TNF-α (Figure 3E), and NO production (Figure 3F). IL-4, an anti-inflammatory cytokine strongly associated with an M2 phenotype was also examined, but neither LPS nor D-mannose at the doses tested yielded reliable IL-4 protein increases in these cells (data not shown). Thus, in cultured macrophage cells during stimulatory conditions that activate inflammatory pathways, D-mannose creates a robust reduction in classic proinflammatory factors while significantly elevating anti-inflammatory IL-10 protein.

While *in vivo* application of D-mannose created a transient pain reversal (Figure 3A and B), we speculate the enduring pain reversal observed in Figure 2 is due in part to a dramatic increase in pDNA-IL-10 efficacy by an action of D-mannose to augment transgene uptake. To directly examine whether an M2-like activation profile is sufficient for improved transgene uptake, IL-4 or D-mannose treatment followed by pDNA-IL-10 uptake in cultured RAW 264.7 macrophages (mouse macrophage cell line) was quantified. Cells were incubated with either IL-4 (100 ng) or D-mannose (500 mM), pDNA-IL-10 (encoding rat IL-10 protein; 0.5 μg or 5.0 μg), IL-4 + pDNA-IL-10 (0.5 μg or 5.0 μg), or D-mannose (500 mM) + pDNA-IL-10 (0.5 μg or 5.0 μg). Compared to untreated controls, IL-4 or pDNA alone treatment, IL-4 revealed no improvement in IL-10 transgene uptake (Figure 3G). However, with D-mannose alone or pDNA-IL-10 alone, a significant increase in rat IL-10 protein levels was measured when cells were treated with D-mannose in combination with 0.5 μg pDNA-IL-10 (Figure 3H). Moreover, a six-fold greater level of rat IL-10 protein was evident when cells were treated with D-mannose in combination with a ten-fold higher pDNA-IL-10 dose (5.0 μg pDNA-IL-10; Figure 3H). Thus, IL-10 protein levels increased with increased dosages of the pDNA-IL-10 transgene only in the presence of D-mannose. It is important to note that the ELISA for rat IL-10 does not cross-react with mouse IL-10 protein levels, demonstrating that plasmid-derived IL-10 protein is robustly expressed when the IL-10 transgene is delivered to RAW 264.7 cells in combination with D-mannose.

### I.t. D-mannose pretreatment results in increased IL-10 and decreased IL1-β expression in spinal cord and DRG following i.t pDNA-IL-10

**Figure 4:**
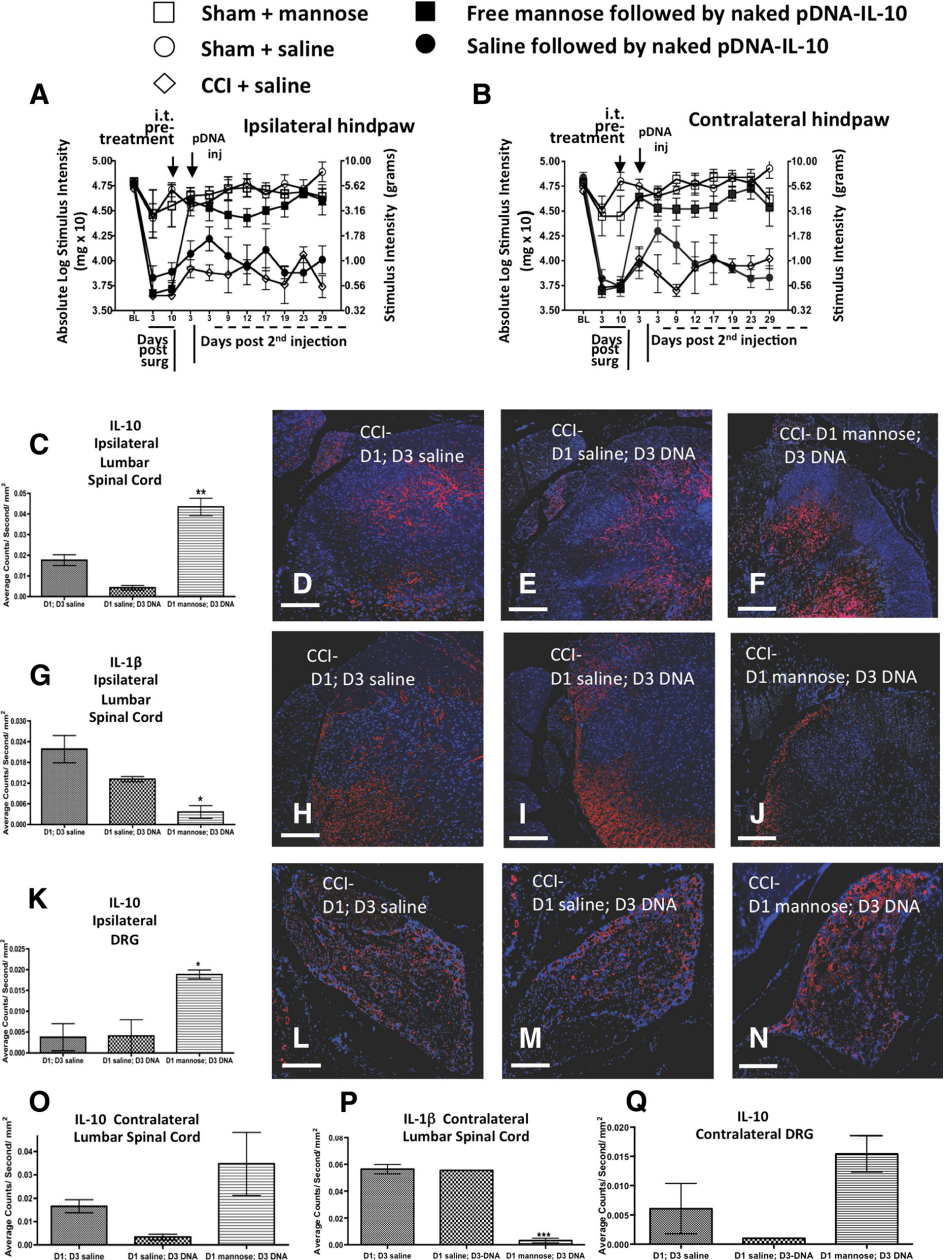
**Spinal and dorsal root ganglia (DRG) pro-and anti-inflammatory markers expression. (A and B)** All rats reveal similar BL threshold values. After CCI, bilateral allodynia compared to sham treatment is observed. On day 10, i.t. pretreatment with D-mannose (50 μg) or equivolume saline followed three days later by i.t. pDNA-IL-10 (25 μg; closed squares; n = 7) or equivolume saline (closed circles; n = 4) is given. Saline-CCI treated animals (open diamonds; n = 3) remained allodynic compared to sham-D-mannose (open squares; n = 3) or sham-saline (open circles; n = 6) treated animals (ipsilateral, F(2,9) = 28.79, *P* <0.0001; contralateral, F(2,9) = 19.7, P < 0.001). Pretreatment with D-mannose (50 μg) followed by pDNA-IL-10 (25 μg) reversed bilateral allodynia compared to CCI-control groups (ipsilateral, F(4,126) = 30.27, *P* < 0.0001; contralateral, F(4,126) = 35.75; *P* < 0.0001). **(C)** Day 29 immunohistochemical examination revealed increased IL-10 immunoreactivity (IR) in lumbar spinal cord with D-mannose (50 μg) pretreatment followed by pDNA-IL-10 compared to non-mannose treated control groups (F(2, 5) = 34.23, *P* < 0.01). **(D-F)** Corresponding fluorescent images of the analyzed data (red = IL-10, blue = cell nuclei). **(G)** Decreased IL-1β IR in the ipsilateral lumbar spinal cord compared to non-mannose treated control groups (F(2,5) = 10.67, *P* < 0.05). **(H-J)** Corresponding fluorescent images (red = IL-1β, blue = cell nuclei). **(K)** Increased DRG IL-10 IR in rats pretreated with D-mannose compared to non-mannose treated control groups (F(2, 5) = 10.35, *P* < 0.05). **(L-N)** Corresponding fluorescent images (red = IL-10, blue = cell nuclei). **(O, Q)** Contralateral spinal cord and DRG reveal elevated IL-10 IR, while **(P)** complete IL-1β IR suppression in the contralateral spinal cord is observed following D-mannose pretreatment. *P < 0.05; **P < 0.01; ***P < 0.0001; images 10X; scale bar = 100 μm.

We next examined whether cytokine expression levels critical for pathological pain processing change in spinal regions where active glial and immune cells are found. We examined by immunohistochemical detection, IL-10 and IL1-β immunoreactivity in spinal lumbar and DRG tissue from behaviorally verified rats. There were no differences between groups at BL prior to CCI or sham surgery (ipsilateral, F_(4,18)_ = 0.2597, *P* > 0.89; contralateral, F_(4,18)_ = 0.4947, *P* > 0.93). As before, CCI treated animals revealed clear bilateral allodynia by day 10 compared to sham controls (ipsilateral, F_(4, 18)_ = 35.54; *P* < 0.0001; contralateral, F_(4, 18)_ = 35.96, *P* < 0.0001) (Figure 4A and B). As before, sham or CCI treated animals received an i.t. injection of saline, or D-mannose (50 μg). Compared to sham animals treated with saline or D-mannose, CCI animals given only saline remained allodynic throughout the 29-day timecourse. However, compared to CCI animals given saline followed by pDNA-IL-10 (25 μg) that remained fully allodynic throughout the time period, CCI animals given D-mannose followed by pDNA-IL-10 revealed full reversal from allodynia by day 3 after i.t. D-mannose and remained fully reversed through 29 days following i.t. pDNA-IL-10 (Figure 4A and B). Duplicating the results of our prior experiment above, i.t. D-mannose followed three days later by i.t. pDNA-IL-10 produced full and sustained reversal from allodynia compared to controls. On day 29 following behavioral assessment, L5 level spinal cord was collected, an anatomical region that receives information from the centrally projecting terminals of damaged sciatic nerve fibers entering the spinal cord. Additionally, L5 DRG were collected, which contain the neuron cell bodies of the associated sciatic nerve axons.Three groups of behaviorally verified rats (data shown in Figure 4A and B) were selected for analysis of IL-10 and IL-1β immunoreactive (IR) bilateral spinal and DRG expression. All tissue sections were collected from CCI treated rats given: (1) a double i.t. saline injection with rats revealing enduring allodynia, (2) i.t. saline followed three days later by i.t. pDNA-IL-10 with rats similarly showing sustained allodynia, and (3) i.t. D-mannose followed three days later with i.t. pDNA-IL-10. Notably, this third group revealed robust reversal from allodynia throughout the timecourse. Thus, for immunohistochemical (IHC) analysis, an examination was undertaken of whether the behavioral profile supports D-mannose pretreatment as a critical factor for improving IL-10 transgene expression as measured by corresponding increases in IL-10 and decreases in IL-1β immunoreactivity. Primary antibodies IR for IL-10 and IL-1 were detected using IR Rhodamine Red fluorophore-conjugated secondary antibodies. Immunofluorescence was quantified using computer-assisted Nuance spectral analysis software as described in the methods.Analysis of spinal cord for IL-10 IR revealed D-mannose pretreatment followed by pDNA-IL-10 injection resulted in significant increases in ipsilateral IL-10 expression compared to saline non-mannose treated controls, with similarly strong trends in the contralateral spinal cord (Figure 4C and O). Representative photomicrographs of the ipsilateral quantified data are shown (Figure 4D-F) revealing spinal IL-10 IR occurs primarily throughout the gray matter. In the DRG, similar bilateral increases in IL-10 IR were observed from rats treated with D-mannose-pDNA-IL-10 compared to saline only or saline followed by pDNA-IL-10 treatment (Figure 4K and Q). Representative ipsilateral photomicrographs (Figure 4L-N) are shown. Conversely, bilateral spinal IL-1β IR is significantly reduced in D-mannose pretreated pDNA-IL-10 injected rats compared saline treated controls (Figure 4G and P). Representative photomicrographs from ipsilateral tissue are shown (Figure 4H-J), which depict a punctate pattern primarily in the white matter, and to a lesser extent, within dorsal spinal cord in deeper laminae.

Given that high variance was observed only in the saline-pDNA-IL-10 group, sample criteria for the detection of outlying observations were applied [60,61] (as described in Methods) to identify whether a true outlier from the group mean existed. One animal was omitted from the lumbar and DRG analyses, resulting in saline-pDNA-IL-10 data that is representative of eight, and not twelve, tissue slices.

### A single i.t. co-injection of D-mannose with a very low dose of pDNA-IL-10 extends reversal of allodynia

**Figure 5:**
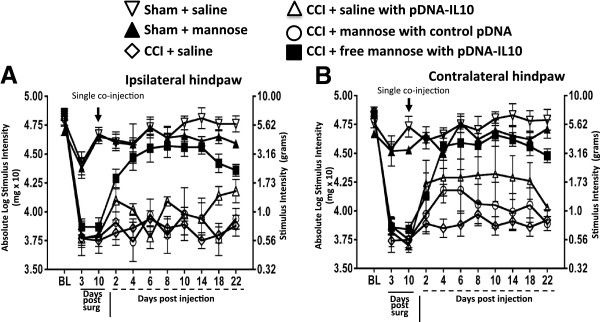
**A single co-injection of D-mannose with low dose pDNA-IL-10 produces enduring reversal of allodynia. (A and B)** after baseline (BL) assessment, bilateral hindpaw responses from sham versus chronic constriction injury (CCI) treated animals revealed bilateral allodynia through day 10 (ipsilateral, F_(4,32)_ = 32.07, *P* < 0.0001; contralateral, F_(5, 32)_ = 38.78; *P* < 0.0001). After testing on day 10, sham rats received a single i.t. saline injection (open inverted triangles; n = 8) or D-mannose (50 μg; closed triangles; n = 4), and CCI rats received a single i.t. co-injection of: (1) saline alone (open diamonds; n = 6), (2) equivolume saline with pDNA-IL-10 (1 μg; open triangles; n = 3), (3) D-mannose (50 μg) with ‘control pDNA’, which lacks the *IL-10* gene (control pDNA; open circles; n = 7), or (4) D-mannose with pDNA-IL-10 (closed squares; n = 10). Sham treated groups (saline or D-mannose) and CCI-saline, or CCI-D-mannose with control pDNA resulted in no change from allodynia while D-mannose co-injected with of pDNA-IL-10 (1 μg) resulted in full reversal of allodynia throughout the three-week time course (ipsilateral F_(5, 238)_ = 60.23; *P* <0.001; contralateral F_(5, 224)_ = 22.18, *P* < 0.001).

The above data reveal robust anti-inflammatory responses of macrophage cells in culture following D-mannose exposure suggesting that D-mannose may act to shift a discrete population of macrophages to a pro-phagocytic anti-inflammatory phenotype. Additionally, within a 24 hour period, *in vivo* i.t. D-mannose pretreatment induces a dramatic anti-inflammatory spinal phenotype, as observed by enduring pain reversal (Figures 2 and 4A and B). These data strongly suggest that D-mannose can act to shift local peri-spinal immune cells to an anti-inflammatory phenotype within a 24 hour period. To expand upon these findings, we examined whether the efficacy of a very low dose of pDNA-IL-10 (1 μg) shown to induce an approximately nine-day pain suppression (Figure 2) could be improved by a single co-injection of D-mannose to induce a long-duration multi-week pain reversal. No significant hindpaw BL differences were observed between groups prior to CCI or sham surgery (ipsilateral, F_(5, 32)_ = 0.8932, *P* > 0.49; contralateral, F_(5, 32)_ = 1.393; *P* = 0.231). As before, CCI treated animals revealed clear bilateral allodynia through day 10 compared to sham controls (ipsilateral, F_(4,32)_ = 32.07, *P* < 0.0001; contralateral, F_(5, 32)_ = 38.78; *P* < 0.0001). On day 10, sham controls given i.t. saline or D-mannose remained non-allodynic throughout the timecourse compared to CCI animals given i.t.: (1) saline alone, (2) saline + pDNA-IL-10, or (3) D-mannose + control pDNA (non-coding pDNA; identical construct minus the *IL-10* gene) (Figure 5A and B). However, animals that received a single i.t. co-injection of D-mannose (50 μg) with pDNA-IL-10 (1 μg) resulted in a clear reversal of allodynia that was sustained for 26 + days (Figure 5A and B). Thus, a single injection of D-mannose (50 μg) co-injected with pDNA-IL-10 (1 μg) reverses allodynia for more than three weeks.

## Discussion

The studies in the current report set out to determine whether M2 activation was sufficient to generate enhanced spinal transgene uptake and instead demonstrated that some factors classically known to induce an M2 phenotype were unable to improve transgene expression. The current report has, however, identified a transgene adjuvant which induces a pro-phagocytic anti-inflammatory milieu, thereby improving non-viral naked pDNA uptake and transgene therapeutic efficacy. The timing and dose refinement of peri-spinal intrathecal (i.t.; subarachnoid) pDNA therapy is achieved with i.t. pretreatment of the small molecule, D-mannose, described herein. Prior reports demonstrate that long-term efficacy of i.t. naked DNA encoding the anti-inflammatory cytokine IL-10 requires pretreatment with pDNA (either encoding or not encoding IL-10) or the immune stimulatory molecule oligodeoxynucleotide (ODN) [24]. The potential actions of peri-spinal immune stimulators were thought to be advantageous because macrophages and other immune cells are present in peri-spinal CSF [30] or spinal parenchyma [9,10,13,62-64] of neuropathic rats. While i.t. ODN served to prime local innate immune cells for improved uptake of pDNA-IL-10, these ODN adjuvants are characterized to stimulate the immune cell receptor, Toll-like receptor 9 (TLR9) that leads to proinflammatory cytokine production, [65] suggesting that ODNs may not be the ideal adjuvant to optimize transgene uptake [66].

### Lack of effect with dexamethasone

Unlike ODN adjuvants, corticosteroids provide robust anti-inflammatory effects by suppressing the activation of multiple proinflammatory genes [67,68] and thereby, may act to facilitate transgene uptake by controlling pDNA-derived proinflammatory cytokine production. A recent report demonstrated that dexamethasone, a potent synthetic glucocorticoid widely used to control clinical inflammatory conditions [68], co-delivered with pDNA, yielded greater transgene uptake and lower proinflammatory cytokine levels [69]. Surprisingly, results in the current report demonstrate that i.t. pretreatment with dexamethasone followed by i.t. pDNA-IL-10 failed to significantly improve the efficacy of pDNA-IL-10 gene therapy, as demonstrated by unaltered chronic allodynia (sensitivity to light touch) (Figure 1). Curiously, more recent studies demonstrated dexamethasone induced increases in cell motility [70], and increased formation of cross-linked actin network (CLAN) important for interacting with transmembrane bound β3-integrin signaling adhesion molecules [71]. β3-integrin activation is important for migration of fibrinogenic cells [72], and blood-brain barrier disruption can involve fibrinogen-mediated inflammation [73]. Further evidence that dexamethasone can play a role in cell migration was demonstrated by its effects on augmenting the actions of the chemokine receptor, CXCR4 on T cells [74]. A recent study conducted in healthy males revealed elevated inflammatory responses in skeletal and adipose tissue following acute dexamethasone treatment [75]. Given strong evidence exists that peripheral neuropathy in rodent models leads to a weakened blood-spinal barrier with robust chemokine/proinflammatory cytokine signaling and infiltration of activated macrophages [10], it is possible that acute i.t. dexamethasone in the current study exacerbated ongoing cellular influx with a proinflammatory phenotype reducing transgene expression and subsequent suppression of allodynia.

### Enhanced pain reversal effects with D-mannose

Conversely, i.t. pretreatment with D-mannose, also characterized to exert anti-inflammatory effects [76,77], significantly altered the efficacy of a subsequent i.t. pDNA-IL-10 injection in that complete and enduring suppression of bilateral allodynia was observed at pDNA-IL-10 doses five-fold lower than previously documented (Figure 2) [24,46]. In addition, we demonstrate enduring bilateral neuropathic pain suppression following a single i.t. injection of D-mannose in combination with naked pDNA-IL-10 (1 μg total). These experiments support the notion that D-mannose may dramatically alter an active and ongoing process in the peri-spinal microenvironment under neuropathic conditions [20]. While not yet fully investigated *in vivo*, these local changes in immune cell phenotype allow lower doses of i.t. pDNA-IL-10 to be transformed into efficacious treatments for pain suppression.

### D-mannose is anti-inflammatory and augments transgene uptake

The possibility that D-mannose could exert enduring pain reversal in the absence of i.t. IL-10 transgene treatment remained unanswered and was examined herein. Not surprisingly, i.t. D-mannose alone produced acute bilateral dose-dependent reversal (approximately nine days) of allodynia (Figure 3A and B), as mannose receptor activation has been characterized to induce an anti-inflammatory cytokine profile [77-79]. These results additionally suggest that D-mannose alters local crucial mediators of neuropathic pain (proinflammatory factors) for a distinct period of time that encompasses a critical window for optimizing transgene uptake. However, the transient allodynia reversal suggests that the enduring pain suppression observed in Figure 2 is due mainly to peri-spinal pDNA-IL-10 treatment following D-mannose pretreatment and not from the effects of D-mannose alone.

In further support of prior studies documenting the anti-inflammatory role of D-mannose, the current data show that D-mannose not only blunts the protein release of the proinflammatory cytokine TNF-α, and the inflammatory signaling molecule NO generated by calcium-independent inducible NO synthase, but also fully suppresses IL-1β with simultaneous increases in IL-10 protein levels in lipopolysaccharide (LPS) stimulated macrophage cell cultures (Figure 3C-F). Thus, these data suggest two important and related mechanisms could be altered by the spinal application of D-mannose. First, a single injection of D-mannose creates transient but complete reversal of allodynia in an animal model of peripheral neuropathy mediated by TNF-α, IL-1β and NO actions. Thus, D-mannose could exert broad spinal anti-inflammatory actions relevant to pathological pain processing. Second, the spinal anti-inflammatory effects of D-mannose may be critical for inducing enhanced phagocytosis thereby optimizing naked pDNA-IL-10 uptake. Indeed, recent evidence supports effective mannose receptor-mediated non-viral gene delivery as superior to currently available transfectant reagents [80,81].

Given that D-mannose pretreatment profoundly improved pDNA-IL-10 efficacy as demonstrated by a > 90-day pain-reversal profile at transgene doses previously reported as moderately and transiently effective [24,46], we examined whether D-mannose could directly improve transgene IL-10 uptake in cell culture. The data are striking and demonstrate that in the presence of D-mannose, transgene-derived rat IL-10 protein levels increase as the IL-10 transgene dose increases. Surprisingly, IL-4, a classic M2 cytokine completely lacked effects for increased transgene uptake suggesting that the actions of D-mannose extend beyond simply inducing an M2 phenotype, or, that a number of factors acting in concert are required for observing an M2 phenotype. Thus, *in vivo*, D-mannose may be acting as a complementary factor for enhanced transgene uptake.

### D-mannose augments IL-10 and reduces IL-1β *in vivo*

Bilateral spinal cord and DRG from rats showing enduring bilateral reversal from allodynia given i.t. D-mannose pretreatment followed by i.t. pDNA-IL-10 (Figure 4A and B) revealed bilateral increases in IL-10 IR. While spinal IL-10 increases contralateral to the CCI did not reach statistical significance (*P* = 0.09), these data suggest that pretreatment with D-mannose improves transgene uptake for sustained pain suppression acting as a complementary factor for stimulating peri-spinal immune cells to respond to transgene material. Increased IL-10 may itself generate an anti-inflammatory pro-phagocytic phenotype [81-84].

Simultaneous and significant bilateral decreases in IL-1β IR are observed (Figure 4C-N) in the spinal cord and in corresponding L5 DRG. Staining is observed in the white matter. Prior reports reveal a similar pattern reflecting expression in oligodendrocytes as previously described [85,86]. The DRG is home to sensory neurons and i.t. injection of D-mannose followed by pDNA-IL-10 three days later within the sensitization period alters DRG cellular IL-10 IR expression levels compared to experimental groups without D-mannose pretreatment. The bilateral DRG expression of IL-10 is very similar to that previously reported by our laboratory [55,56]. Our prior reports indicate IL-10 is expressed by satellite glial cells and/or infiltrating macrophages capable of modifying sensory neuron activity and subsequent allodynia [55,56]. Thus, D-mannose pretreatment followed by spinal *IL-10* gene therapy generates an effective and long-duration anti-inflammatory local spinal and DRG environment and these changes generate enduring IL-10 transgene induced pain suppression.

### The period of sensitization

A critical element in the studies described herein is the period of sensitization whereby improved therapeutic transgene uptake occurs (Scheme 1). The period of sensitization can be exploited to achieve a single co-injection consisting of a small-molecule adjuvant in combination with pDNA-IL-10. D-mannose leads to a local anti-inflammatory phenotype similar to that observed in M2 activated immune cells that may only be important during the period of sensitization for efficient non-viral transgene phagocytosis. Indeed, a recent report engineered a mannose-specific targeted non-viral gene delivery method that resulted in superior gene expression in macrophage cell lines [80]. The data reported here show, for the first time, that a previously demonstrated ineffective low dose of non-viral pDNA-IL-10 (1 μg) gene therapy [87] is now efficacious for over three weeks when delivered with mannose upon a single i.t. injection (Figure 5). While D-mannose-IL-10 efficacy in controlling allodynia long after it has been established (for example, 45 or 60 days) could be highly informative as it relates to clinical therapeutic applications, examination of D-mannose-IL-10 gene therapy in newly established allodynia (10 days) was the key focus in this report because enduring transgene efficacy of 90 days was a possibility. Notable, CCI is an animal model characterized as revealing a stable approximately 60-day allodynia, [29,46,88,89].

Prior work demonstrates that relief from allodynia during CCI-induced neuropathy is observed at increasingly longer intervals following each subsequent i.t. pDNA-IL-10 injection, but not with control pDNA. That is, allodynia is reversed 3, 7, and 26 days following an initial, second and third i.t. injection, respectively [29], which supports that (a) the IL-10 receptor is not desensitized following prolonged exposure, and (b) an ongoing active cellular process is present. Adjustment of the time interval between the initial and second pDNA-IL-10 injection, such that the second injection occurs during reversal from allodynia (for example, three-day inter-injection interval), results in an approximately two-month pain relief profile [29]. Further examination revealed the magnitude and the duration of reversal from allodynia is significantly diminished and shortened if the inter-injection interval occurs outside sensitization period (5 to 72 hour) [24]. Together, data from the current and prior reports suggest time-sensitive immune cell specific activation in the lumbar meninges surrounding the i.t. injection site may be beneficially exploitive for therapeutic transgene expression.

### Bilateral allodynia, spinal IL-10 and IL-1β

The observed bilateral behavioral changes that correspond to bilateral cytokine alterations following peripheral neuropathy supports observations from prior reports [29,54-56,90], with other reports demonstrating corresponding changes in glial activation [55,56,91-93]. While neuropathic pain resulting from inflammation and/or trauma to peripheral nerves is most widely characterized as arising from the body region in which the trauma/inflammation occurred, pain may also be perceived as arising from the healthy contralateral (‘mirror-image’) body region [91]. Indeed, a variety of contralateral biochemical changes are observed following unilateral nerve injury [91,94]. The spread of pain from one side of the body to the other may involve a contralateral spread of spinal cord excitation via gap junctions, which are direct, physical links between large populations of astrocytes allowing local astrocyte activation to activate astrocytes at distant, contralateral sites [95]. Moreover, under peripheral neuropathic conditions, TNF-α release into spinal CSF and diffusion to contralateral glial satellite cells results in nerve growth factor mediated bilateral allodynia [91].

The data from the current study further support bilateral spinal glial cytokine actions in producing or inhibiting allodynia in neuropathic rats, as assessed by increased immunofluorescent detection of markers for IL-1β and IL-10. Notably, astrocytes as well as microglia express IL-10 and IL-1β [96] and the mannose receptor [97,98]. While D-mannose pretreatment followed by pDNA-IL-10 abolished bilateral spinal cord IL-Iβ IR, it significantly increased ipsilateral IL-10 IR in both the spinal cord and the DRG, while generating a strong trend toward IL-10 IR in the contralateral spinal cord and DRG. Microglia may be a source of IL-10 production, as bilateral microglial activation is concurrent with bilateral reversal from allodynia [55,56]. IL-10 is a pleiotropic cytokine, which acts as a negative regulator to a variety of proinflammatory factors [17].

While not examined in the current report, the anti-inflammatory cytokine, Transforming Growth Factor-beta1 (TGF-β), controls chronic peripheral neuropathy [10,99] and may be an additionally important factor mediating an anti-inflammatory environment with subsequent transgene uptake following D-mannose pretreatment. The bilateral decrease in IL-1β IR in the spinal cord upon D-mannose pretreatment supports prior work that significantly reduced bilateral IL-1β expression occurs concurrently with allodynia reversal [55,56]. Therefore, mannose pretreatment, rather than pDNA-IL-10 alone, is important in generating sufficient spinal IL-10 transgene expression to inhibit bilateral IL-1β expression and bilateral allodynia.

### Allodynia and thermal hyperalgesia

One advantage of utilizing CCI-induced neuropathy is that while bilateral allodynia is observed, unilateral hindpaw thermal hyperalgesia on the side ipsilateral to the nerve injury is well-documented, with this model of neuropathy widely used since its first report over 24 years ago [53]. Thermal hyperalgesia is mediated by A-delta and C fiber nociceptors and is assessed by examining exaggerated responses to nociceptive input. Moreover, nociceptors can become sensitized through a process of peripheral sensitization [100]. However, bilateral allodynia, mediated by non-nociceptive A-beta fibers, involves spinal mechanisms [100,101], and is a reliable consequence of sciatic nerve CCI in the rodent model [88] applied in the current report. Underlying mechanisms of chronic neuropathy can be gleaned when nociceptors and A-beta fiber sensory responses are altered [101,102] providing insight into new therapeutic targets. For example, i.t. application of pDNA-IL-10 gene therapy reveres chronic hyperalgesia induced by CCI [103] and allodynia, suggesting that spinal *IL-10* gene therapy could be a viable treatment option for individuals refractory to currently available pain therapeutics. Given the potential clinical applications of non-viral spinal gene therapy, allodynia was the sole endpoint examined in the current report because neuropathic allodynia is a major common problem in chronic pain patients [104].

### The local peri-spinal meningeal environment

Leukocytes like macrophages and dendritic cells are the predominant immune cells found in the meninges, including the pia mater, which is in direct contact with underlying spinal cord pain-projection neurons [13,105]. Increases in these cell types in meninges after chronic neuropathic pain produced by partial sciatic nerve ligation have been identified [13], suggesting that these leukocytes may make up a population of sensitized cells upon D-mannose treatment leading to subsequent pDNA uptake within the sensitization interval. Notably, additional local macrophage cell enrichment measured in CSF from hours to days after an i.t. pDNA-IL-10 injection corresponds to the sensitization interval [24,30].

### Applications as a prophylactic treatment

The observation that i.t. pDNA-IL-10 in healthy, non-neuropathic control groups results in only small increases in IL-10 transgene expression in spinal meninges and DRG [22] suggests that conditions in non-pathological spinal cord are not optimal to generate substantial pDNA-based IL-10 transgene expression. Indeed, pain is only minimally alleviated when i.t. pDNA-IL-10 is injected prior to full allodynia [22]. We speculate that pre-emptive i.t pDNA-IL-10 is minimally effective because the local environment may not be permissive (for example, insufficient leukocyte accumulation) for non-viral transgene uptake and subsequent expression. Clinically, gene delivery-based therapeutics will be applicable to people who have persistent pain, and less so for prophylactic pain treatment. Together, these data suggest that infiltrating leukocytes localize to the peri-spinal meninges and participate in active and ongoing cellular processes under neuropathic conditions. Exploiting the diseased condition may impact continuous transgene expression and efficacy.

### D-mannose

D-mannose and associated mannose receptor have interesting anti-inflammatory properties. D-mannose is a simple hexose sugar with a molecular weight of 180.2 and is known to: decrease inflammatory processes during wound healing [76], reduce oxidative bursts required during inflammation [106], suppress adjuvant-induced arthritis in a rat model [107], inhibit LPS-induced IL-1β, TNF-α decrease NF-kB/p65 critical for proinflammatory cytokine expression, and decrease leukocyte influx following intratracheal instillation of LPS, which is a model of sepsis-associated acute lung injury and respiratory distress syndrome [77,108].

### The mannose receptor (MR)

The MR is a transmembrane glycoprotein pattern recognition receptor (for review of structure, see [109]) involved in host defense of innate immunity by recognizing mannosylated ligands (for example, lysosomal hydrolases) that can include a variety of bacteria, yeasts and parasites expressing mannosylated molecules [110-113], which critically mediates pinocytosis and phagocytosis. In the rodent CNS, the MR is expressed throughout the brain and brainstem in neurons, microglia, astrocytes, and in the associated glia limitans that is made up of astrocyte endfeet [97]. In some brain regions, MR expression diminishes with age revealing clear expression differences in adults (for example, basal ganglia, and thalamus) while other regions, including the glia limitans and cerebellar neurons, reveal persistent MR expression.

In the spinal cord, MR expression is primarily observed in the glia limitans, leptomeninges (pia and arachnoid mater) and in perivascular microglia [114] in adult rodents. Following spinal cord injury, the MR and other M2 polarized indicators like arginase-1, IL-4, IL-10 and IL-13 are increased [115-117]. As such, an additional role for the MR is homeostatic clearance of endogenous molecules that are up-regulated during inflammation [118,119]. Thus, the spinal expression profile of the MR occurring in the leptomeninges and perivascular microglia under normal conditions, with increased expression following inflammatory processes, provided the logical basis in the current report for administering D-mannose intrathecally, as this anatomical region is made up of the leptomeninges and underlying glia limitans. Moreover, i.t. administration following chronic allodynia provides a primed proinflammatory environment for D-mannose to act at the MR. We previously demonstrated robust transgene expression following 100-fold higher doses of pDNA (without adjuvant co-administration), in the meninges surrounding the i.t. injection site [29]. Improved efficiency of peri-spinal non-viral gene delivery can be achieved following increased MR activation that generates enhanced phagocytic ability in the presence of anti-inflammatory factors. Indeed, mannose-mediated MR activation leads to secretion of IL-10 and other cytokines that contribute to a down-regulation of proinflammatory immune responses including decreased IL-1β and TNF-α protein production [78]. The data reported here are in support of these prior reports and extend these findings by demonstrating that peri-spinal mannose stimulates an anti-inflammatory profile both *in vitro* and *in vivo*.

While examination of spinal MR expression concurrent with reversal from allodynia could provide some insight into the spinal cord milieu underlying pain reversal, it is also possible that no change in MR expression levels could occur. One reason is that a multitude of stimuli are present in the spinal environment including evidence for activated microglia, astrocytes and perivascular macrophages expressing anti-inflammatory factors [120]. Moreover, infiltrating leukocytes such as macrophages and T cells can occur, and ongoing cellular turnover could obscure shifting MR-expressing cells, while anti-inflammatory factors like IL-10 remain elevated. Together, these cellular and biochemical events at different magnitudes in the spinal cord during a prolonged allodynia reversal (30 to 90 days) may diminish the predictability of, or may not align with, spinal MR expression occurring concurrently with pain reversal. While speculative, MR expression levels may not directly follow pain profiles.

## Conclusions

The current data show the effects of D-mannose during the sensitization period, defined as a local peri-spinal cellular response that occurs during a three-day inter-injection interval. D-mannose significantly improves peri-spinal non-viral transgene uptake during this period that is followed by enduring pain suppression of over 90 days in a rat model of peripheral neuropathy. *In vitro* and *in vivo* examination of proinflammatory and anti-inflammatory factors suggest that D-mannose stimulates a local cellular response that is both anti-inflammatory and phagocytogenic, and in doing so, exploits a response that eliminates the necessity of a second pDNA-IL-10 injection. In this report, reducing injection numbers to a single co-injection of D-mannose with free pDNA-IL-10, at doses ten-fold lower than previously reported, remains robustly efficacious leading to a stable suppression of allodynia. It is possible that mannose acts to ‘stimulate’ cells in the meninges, thus augmenting glial and innate immune phagocytosis of pDNA-IL-10. Future non-viral transgene carrier formulations, aimed at protecting pDNA degradation and that include D-mannose, could further optimize early transgene uptake resulting in pain control of unprecedented duration. These data additionally support the potential application of D-mannose-enhanced non-viral IL-10 therapy that extends beyond neuropathic pain treatment to the treatment of degenerating CNS disease conditions.

## Abbreviations

ANOVA: analysis of variance; bp: base pair; BL: baseline; BSA: bovine serum albumin; CCI: chronic constriction injury; CLAN: cross-linked actin network; CNS: central nervous system; CSF: cerebrospinal fluid; DAPI: 4′,6-diamidino-2-phenylindole; DEX: dexamethasone; DMEM: Dulbecco’s modified Eagle’s medium; DPBS: Dulbecco’s phosphate-buffered saline; DRG: dorsal root ganglia; ELISA: enzyme-linked immunosorbent assay; i.t.: intrathecal; FBS: fetal bovine serum; IACUC: Institutional Care and Use Committee; IHC: immunohistochemical; IL-1β: interleukin-1β; IL-4: interleukin-4; IL-10: interleukin-10; IR: immunoreactive; LCTF: liquid crystal tunable filter; LPS: lipopolysaccharide; M1: classical activation; M2: alternative activation; MR: mannose receptor; NDS: normal donkey serum; NO: nitric oxide; ODN: oligodeoxynucleotide; TNF-α: tumor necrosis factor-alpha; PBS: phosphate-buffered saline; pDNA: plasmid DNA; pDNA-IL-10: plasmid DNA encoding the *IL-10* gene; ROI: region of interest; TGF-β: Transforming Growth Factor-beta1.

## Competing interests

The authors report no competing interest.

## Authors’ contributions

ECD designed the research, analyzed the data and wrote the paper; LAA, BNB, and DRM were critically involved in the behavioral data collection and analysis; AAK performed all aspects of the *in vitro* cell culture research and wrote the relevant methods and results sections, JLW collected and analyzed the spinal tissue for immunohistochemical analysis; EL performed injections and surgical manipulations; JAW provided guidance on immunohistochemical procedures and manuscript preparation; and EDM designed all research experiments, assisted in data collection, analysis and interpretation, and wrote the manuscript. All of the authors have read and approved the final version of the manuscript.
